# Risk factors for depression during the COVID-19 pandemic: a longitudinal study in middle-aged and older adults

**DOI:** 10.1192/bjo.2021.997

**Published:** 2021-09-02

**Authors:** Jamie Rutland-Lawes, Anna-Stiina Wallinheimo, Simon L. Evans

**Affiliations:** Faculty of Health and Medical Sciences, University of Surrey, UK; Faculty of Health and Medical Sciences, University of Surrey, UK; Faculty of Health and Medical Sciences, University of Surrey, UK

**Keywords:** COVID-19, older adults, depression, mental health, social isolation

## Abstract

**Background:**

The COVID-19 pandemic and resultant social restrictions have had widespread psychological ramifications, including a rise in depression prevalence. However, longitudinal studies on sociodemographic risk factors are lacking.

**Aims:**

To quantify longitudinal changes in depression symptoms during the pandemic compared with a pre-pandemic baseline, in middle-aged and older adults, and identify the risk factors contributing to this.

**Method:**

A total of 5331 participants aged ≥50 years were drawn from the English Longitudinal Study of Ageing. Self-reported depression symptoms in June/July 2020 were compared with baseline data from 2–3 years prior. Regression models investigated sociodemographic and lifestyle variables that could explain variance in change in depression.

**Results:**

Within-participant depression scores increased significantly from pre-pandemic levels: 14% met the criteria for clinical depression at baseline, compared with 26% during the pandemic. Younger age, female gender, higher depression scores at baseline, living alone and having a long-standing illness were significant risk factors. Gender-stratified regression models indicated that older age was protective for women only, whereas urban living increased risk among women only. Being an alcohol consumer was a protective factor among men only.

**Conclusions:**

Depression in UK adults aged ≥50 years increased significantly during the pandemic. Being female, living alone and having a long-standing illness were prominent risk factors. Younger women living in urban areas were at particularly high risk, suggesting such individuals should be prioritised for support. Findings are also informative for future risk stratification and intervention strategies, particularly if social restrictions are reimposed as the COVID-19 crisis continues to unfold.

The COVID-19 pandemic has had significant psychological, financial and social ramifications for populations around the world. Despite affecting individuals from all age groups, adults aged ≥50 years have been disproportionately affected in terms of hospital admission and mortality risk.^1^ The UK Government implemented a national ‘lockdown’ on 23 March 2020, to reduce the spread of COVID-19. Under lockdown restrictions, all unnecessary social contact ceased and nonessential businesses closed, and individuals were told to remain at home and could only leave to purchase essentials and seek emergency medical attention. Despite the necessity of lockdown, it has had significant consequences for psychological well-being.^2^ Older adults, particularly those living alone, experience greater social isolation and loneliness^3^ compared with younger age groups. Social isolation has been consistently identified as a key factor for depression risk in older adults.^4^ The social restrictions during lockdown thus have the potential to particularly affect the mental well-being of older age groups.^5,6^ Social isolation has been associated with psychological distress across the lifespan;^7^ during the COVID-19 pandemic, positive associations have been found between social isolation, psychological distress and mental health issues among adults aged ≥55 years.^8,9^ However, to date, work investigating the mental health impact of lockdown has tended to focus more on younger age ranges, or recruit participants from across the adult age range. Information on effects specific to middle-aged and older adults is lacking.

Despite some evidence suggesting that older age groups have proved more resilient than initially feared,^10^ determining the factors placing these individuals at higher risk of depression during lockdown should be prioritised.^6^ This would help ensure that the groups most at risk receive the support they need, and mitigate the ongoing mental health impact on adults at middle-aged and above.

## Impact of lockdown across the lifespan

There is very limited work focusing on depression in middle-aged and older adults during lockdown. Almost all studies to date have considered individuals from across the adult age range;^11^ mosthave been cross-sectional,^12^ and very few have been longitudinal studies.^13,14^ Collectively, these studies point to risk factors that include being female, living alone, having pre-existing physical or mental health conditions, and lower education and socioeconomic status. Amongst the few longitudinal studies conducted, Daly et al^13^ only measured general changes in mental health, using the General Health Questionnaire-12 (a composite questionnaire assessinganxiety, depression, social dysfunction and loss of confidence), while O'Connor et al^14^ only compared changes in depression symptoms as lockdown progressed, with no comparison made to a pre-pandemic baseline.

## Impact of lockdown on older adults

Regarding middle-aged and older adults, verylimited work has been published to date. Robb et al^4^ conducted a cross-sectional study in UK adults aged ≥50 years, between May and July 2020, to explore factors influencing mental health and well-being at this time point. The findings indicated that women, those who were single/widowed/divorced, those living alone and those reporting feelings of loneliness were more likely to score higher on the depression component of the Hospital Anxiety Depression Scale. There were also gender-specific effects: smoking was linked to higher depression scores in men only, whereas alcohol use raised risk only in women. Interestingly, the study also found an age effect, with older participants in their sample reporting fewer depression symptoms, suggesting that older adults might be more resilient to depression during lockdown compared with those in their 50s. However, because the study was cross-sectional, only limited inferences can be made from these data.

There is an absence of longitudinal studies on the risk factors for depression during the pandemic among middle-aged and older adults. Longitudinal designs that make comparisons with pre-lockdown data are more powerful as changes in depression symptoms can be quantified, and pre-existing depression accounted for. The aforementioned studies point to factors including gender, living alone, socioeconomic status and health status/behaviours as potentially important variables affecting mental health vulnerability during the pandemic, but longitudinal analyses would provide better insights into these, and help identify at-risk groups that should be prioritised for support.

## Aims

To address this important question, we explored which factors increased the risk of depression symptoms during the pandemic, making use of longitudinal data collected by the English Longitudinal Study of Ageing (ELSA).^15^ By comparing data from two time points (June/July 2020 against data from 2–3 years prior), we aimed to establish, using regression models, the sociodemographic and lifestyle factors that influenced within-participant change in depression symptoms from before to during the pandemic. A range of pertinent sociodemographic and lifestyle factors were entered as predictors in the models. Gender-stratified analyses were then conducted to identify gender-specific risk factors.

## Method

### Data and sample

Data for the present investigation are drawn from ELSA. ELSA collects data every 2 years from a representative sample of adults aged ≥50 years who are living in private households in England, the most recent being June 2018 to May 2019 (see Steptoe et al^15^ for further details). ELSA conducted an additional wave of collection within the cohort (the COVID-19 substudy) in June and July 2020 (when lockdown conditions were in force in the UK), specifically to investigate the effects of the pandemic on older people, gathering data on depression symptoms and various lifestyle and health measures. ELSA data is publicly available at https://beta.ukdataservice.ac.uk/datacatalogue/series/series?id=200011.

### Ethical approval

The authors assert that all procedures contributing to this work comply with the ethical standards of the relevant national and institutional committees on human experimentation and with the Helsinki Declaration of 1975, as revised in 2008. All ELSA procedures were approved by the Multicentre Research and Ethics Committee (approval number MREC 01/2/91), and informed consent was obtained from all participants.

### Procedures

The data were accessed under project number 206540. In this study, only participants who completed the COVID-19 substudy were included, and their data from the previous wave (wave 9, June 2018 to May 2019) was also used to provide some of the sociodemographic data that was not collected at the COVID-19 wave and as a baseline for quantifying change in depression during lockdown. In the ELSA substudy, data collection was either by telephone or via internet. All ELSA participants were initially invited to take part (by post) and directed to the online survey (lasting for approximately 30 min). There was a financial incentive of £10 to take part. The NatCen (National Centre for Social Research) telephone unit carried out a computer-assisted telephone interview for those participants who preferred to take part via telephone.

In the current study, the following inclusion criteria were applied: aged ≥50 years and currently living in a private household (i.e. not in care/hospital).

### Depressive symptoms and depression

Depressive symptoms and depression were measured with the seven-item Center for Epidemiologic Studies Depression Scale (CES-D) Short-Form (CES-D-SF). The CES-D-SF asks whether the following symptoms were experienced in the preceding week: depression, sadness, lack of happiness, loneliness, sleep was restless, inability to get going, lack of energy and that everything felt like an effort. The participant responded either yes or no to each (1 point for each), yielding a total score between 0 to 7. The CES-D-SF has psychometric properties comparable to the CES-D; internal and test–retest reliability has been shown. A cut-off based on a total score of ≥3 on the CES-D-SF shows good correspondence with an interview-based clinical diagnosis of depression.^16^ We calculated the change between each individual's CES-D-SF scores (acting as our dependent variable) by subtracting the CES-D-SF total scores recorded in the COVID-19 substudy (lockdown) from those in wave 9 (baseline), to longitudinally quantify the change in depression symptoms in each participant.

### Data analysis plan

Only participants with data on all the variables under investigation were included. We excluded 1063 participants because of missing data (e.g. net financial wealth), yielding a final sample of 5331. Multiple linear regression were employed within the SPSS (version 25 for Windows) general linear model framework, to determine which predictors could explain variance in the dependent variable (change in depression scores between lockdown and wave 9).

We included the following as predictors in the regression models: age; gender; self-reported long-standing illness, disability or infirmity (yes/no); CES-D-SF depression score at wave 9; education (four levels: degree level, higher education, secondary school and below secondary school); number of people in the household (coded as living alone or not alone); ethnicity (non-Black, Asian and minority ethnic (BAME) or BAME); urban/rural living; net financial wealth (total net non-pension household wealth: financial, physical and housing wealth, minus debts, coded by the ELSA study team into tertiles of low, medium or high); current employment status (four levels: employed, unemployed, retired or long-term sick); smoking status (smoker/non-smoker) and alcohol use (drinker/non-drinker). Data for these were drawn from the COVID-19 substudy wave (June and July 2020), apart from education, net household wealth and wave 9 depression scores, which were drawn from ELSA wave 9 (conducted June 2018 to May 2019). All data were based on self-report, apart from urban/rural living, which was based on the Department for Environment, Food and Rural Affairs urban/rural indicator, using the participant's postcode.

We conducted two additional regression analyses, stratified by gender, to investigate gender-specific predictors. Furthermore, to investigate a possible age×gender interaction, a separate 2 (gender: male or female) × 4 (age: decade 1, decade 2, decade 3 and decade 4) between-groups analysis (ANOVA) was conducted, with the change in depression scores as a dependent variable.

## Results

### Descriptive statistics

Data were obtained for 5331 participants (mean age 70.25 years, s.d. 9.30). Women represented 57% of the sample. Table 1 summarises the characteristics of the whole sample. At baseline (wave 9), the mean depression score was 1.09, and 14% of the sample met the criteria for clinical depression based on a cut-off score of ≥3 on the CES-D-SF. At lockdown, mean depression score was 1.65, and 26% of the sample met the criteria for clinical depression. Within-participant comparisons (repeated-measures ANOVA) showed a significant increase in depression scores at lockdown compared with baseline (*F*(1, 6394) = 622.31, *P* < 0.001).

**Table 1:** Summary of participant characteristics (*N* = 5331)

Measure	Mean (s.d.) or %	
CES-D-SF (wave 9)	1.09 (1.49)	
Clinical depression	14%	
CES-D-SF (lockdown)	1.65 (1.94)	
Clinical depression	26%	
CES-D-SF (lockdown – wave 9)	0.56 (1.78)	
Increase in clinical depression	12%	
Age, years	70.25 (9.30)	
Gender	Male: 43%	Female: 57%
Urban/rural living	Urban: 73%	Rural: 27%
Long-standing illness	Present: 55%	Absent: 45%
Drinker/non-drinker	Drinker: 64%	Non-drinker: 36%
Living alone/not alone	Living alone: 27%	Not alone: 73%
Non-BAME/BAME	Non-BAME: 96%	BAME: 4%
Smoker/non-smoker	Smoker: 7%	Non-smoker: 93%
Education (1–4)		
(1) Degree level	23%	
(2) Higher education	26%	
(3) Secondary school	34%	
(4) Below secondary school	17%	
Net financial wealth (by tertile)		
(Low)	30%	
(Mid)	32%	
(High)	38%	
Employment (1–4)		
(1) Employed	22%	
(2) Unemployed	7%	
(3) Retired	69%	
(4) Other	2%	

Clinical depression was defined as the percentage of individuals scoring ≥3 on the CES-D-SF, indicating clinical depression; CES-D-SF (lockdown – wave 9) = change in depression scores (lockdown scores – wave 9 scores). CES-D-SF, Center for Epidemiologic Studies Depression Scale Short-Form; BAME, Black, Asian and minority ethnic.

### Factors explaining change in depression scores

Multiple linear regression was carried out to determine the effect of the predictors on the change in depression scores (lockdown depression score minus baseline depression score). Assumptions of regression regarding normality were met, and multicollinearity based on the variance inflation factor^17^ was acceptable. The regression model was significant (*F*(54, 5277) = 12.16, *P* < 0.001). The analysis suggested that age, gender, long-standing illness, depression at baseline, being a drinker/non-drinker, living alone/not alone, ethnicity and employment status were all significant predictors of the change in depression scores (Table 2). Higher age, male gender, no long-standing illness, depression at baseline, being a drinker, not living alone, being of BAME ethnicity (versus being non-BAME) and employment status (being employed, unemployed or retired versus being on long-term sick leave) were all protective against increased levels of depression during lockdown. The adjusted *R²* indicated that 10% of the variance in the change in depression scores could be explained by the model.

**Table 2:** Regression model with change in depression scores as the criterion variable (in all participants, men only and women only)

	*F* (all)	*P-*value	*F* (men)	*P-*value	*F* (women)	*P-*value
Constant	0.41	0.52	0.29	0.59	0.55	0.46
Age	1.69	0.005**	0.99	0.49	1.51	0.02*
Gender	19.98	0.001**	−	−	−	−
Urban/rural living	3.61	0.06	0.31	0.58	3.95	0.05*
Long-standing illness	25.24	0.001**	6.43	0.01*	17.81	0.001**
CES-D-SF (wave 9)	534.40	0.001**	128.9	0.001**	380.6	0.001**
Drinker/non-drinker	3.96	0.05*	7.45	0.006**	0.38	0.54
Education (1–4)	0.20	0.90	0.95	0.42	0.52	0.67
Living alone/not alone	52.09	0.001**	9.33	0.002**	46.13	0.001**
Ethnicity	5.99	0.014*	2.11	0.15	4.62	0.03*
Net financial wealth (1–3)	0.67	0.51	0.30	0.74	0.60	0.55
Employment status (1–4)	9.31	0.001**	2.65	0.05*	6.60	0.001**
Smoker/non-smoker	0.10	0.76	0.88	0.35	0.04	0.84

Long-standing illness was defined as long-standing illness, disability or infirmity; education was grouped as degree level (1), higher education (2), secondary school (3) or and below secondary school (4); living alone/not alone was determined by the number of people living at home; ethnicity was grouped as non-BAME or BAME; net financial wealth was determined according to tertiles of net financial wealth (1–3); employment status was grouped as employed (1), unemployed (2), retired (3) or other (including long-term sick) (4). CES-D-SF, Center for Epidemiologic Studies Depression Scale Short-Form; BAME, Black, Asian and minority ethnic.

*P<0.05, **P<0.01.

### Men

The regression model was statistically significant (*F*(53, 2220) = 3.46, *P* < 0.001). Long-standing illness, depression at baseline, being a drinker/non-drinker, living alone/not alone, and employment status were all significant predictors of change in depression scores (Table 2). No long-standing illness, lower depression scores at baseline, being a drinker, not living alone and employment status (being employed or retired versus being on long-term sick leave) were all protective in men. The adjusted *R²* indicated that 5% of the variance in the change in depression scores could be explained by the model.

### Women

The regression model was statistically significant (*F*(53, 3004) = 9.14, *P* < 0.001). The analysis indicated that age, urban/rural living, long-standing illness, depression at baseline, living alone/not alone, employment status and ethnicity were all significant predictors of the change in depression scores for women (Table 2). Higher age, rural living, no long-standing illness, lower depression scores at baseline, not living alone, employment status (being employed or retired versus being on long-term sick leave) and being BAME were all protective factors. The adjusted *R²* indicated that 12% of the variance in the change in depression scores could be explained by the model.

### Additional gender×age analysis

There was a main effect of gender on the change in depression scores during lockdown compared with baseline (*F*(1, 6291) = 11.08, *P* < 0.001). Women showed a greater change (estimated marginal mean (EMM) 0.63, s.e. 0.03) than men (EMM0.47, s.e. 0.03). There was no main effect of age by decade (coded as decade 1: 50–60 years, decade 2: 60–70 years, decade 3: 70–80 years and decade 4: 80–90 years) (*F*(3, 6291) = 1.41, *P* = 0.24). However, there was a significant interaction between gender and age by decade (*F*(3, 6291) = 3.08, *P* = 0.03). There was a significant effect of age for women (*F*(3, 3566) = 3.60, *P* = 0.013), but not for men *(P* > 0.05). For women, depression scores reduced with age (decade 1: EMM0.79, s.e. 0.07; decade 2: EMM0.67, s.e. 0.06; decade 3: EMM0.65, s.e. 0.06 and decade 4: EMM0.42, s.e. 0.09): pairwise comparisons (with Bonferroni correction) identified a significant difference between decade 1 and decade 4 (*P* < 0.001), between decade 2 and decade 4 (*P* = 0.014), and between decade 3 and decade 4 (*P* = 0.025).

## Discussion

The present study investigated factors that predicted an increase in depression symptoms from pre-pandemic to mid-2020, in 5331 UK adults aged ≥50 years. Self-report data collected by the ELSA cohort study in June/July 2020 (when UK lockdown restrictions and social distancing measures were in place) was compared with data from June 2018 to May 2019 (baseline). We quantified the change in depression symptoms between the two time points, and a significant overall increase in depression symptoms was observed in the sample. The percentage of individuals meeting criteria for clinical depression based on the CES-D scale nearly doubled, from 14% at baseline to 26% during lockdown.

We then explored the factors that served as predictors of this change, using multiple regression. We considered age, gender, rural/urban living, pre-existing depression at baseline, employment status, level of education, living alone/not alone, longstanding illness, ethnicity, household wealth, smoking and alcohol usage. The regression model was statistically significant (*P* < 0.001); significant predictor variables were female gender, living alone, higher depression symptoms at baseline, being unemployed owing to long-term sickness, having a long-standing health condition, being non-BAME and being a non-consumer of alcohol. Urban living showed a trend level of significance. Household wealth, level of education and smoking status were not significant predictors in the model.

Given the strong effect of gender identified in the first model, and following the approach of Robb et al,^4^ separate gender-specific multiple linear regressions were then conducted to determine how risk factors varied according to gender. For both genders, models were statistically significant (*P* < 0.001), and (as before) higher depression at baseline, being unemployed owing to long-term sickness, living alone and having a long-standing health condition significantly increased the risk of an increase in depression symptoms during lockdown compared with baseline. In women only, older age acted as a protective factor against increased risk of higher depression during lockdown. Additionally, urban living and non-BAME ethnicity significantly increased risk in women only. In men only, being a drinker of alcohol was a significant (protective) predictor in the model, associated with a lower change in depression scores. Thus, the findings point to gender-specific risks and protective factors in this population.

### Gender

The finding that female gender enhanced risk of depression during lockdown is consistent with other studies on the effects of the pandemic across the adult age range.^4,11^ This has been attributed to gender disparities in wealth, exacerbated by a disproportional economic impact of the COVID-19 pandemic on women, deriving from greater increases in childcare responsibilities, more disruption to paid work in female-dominated job sectors and greater job loss.^18^ However, these factors might be less relevant for the age range under study here. One possible explanation relates to levels of worry, as women have been shown to generally worry more than men.^19^ Women also have a higher likelihood of being diagnosed with depression generally, and this is well established in the literature.^20^ Another factor that might have contributed to the findings, stemming from the self-report nature of the data, could be a possible tendency for male participants to underreport their depression symptoms. However, this is somewhat mitigated by the longitudinal design, which allowed within-participant change to be quantified and used as the dependent variable: this mitigates potential between-gender reporting biases and is a key strength of the study.

### Age

Across the whole sample, age emerged as an important predictor variable for change in depression, in that higher age was protective. This finding accords with previous studies on COVID-19-related effects on mental health in different age groups: younger age groups seem at higher risk of increased depression compared with older adults;^11^ a cross-sectional study by Robb et al^4^ in adults aged ≥50 years also found that younger age in their cohort was associated with higher anxiety and depression scores. One possible explanation for this is enhanced psychological resilience in older adults.^20^ Older adults have also been shown to worry less than younger adults,^19^ and have superior emotion regulation and coping strategies.^21^^,^^22^ Greater resources in older adults (such as higher social status and financial stability) could also contribute to better resilience with age.^10^

However, the gender-stratified analyses indicated that higher age was protective in women only, and this was confirmed by a significant interaction effect between gender and age when grouped by decade. This is a novel finding in COVID-19-related mental health studies, but is in accordance with epidemiological studies conducted before the pandemic, showing that women aged <65 years have a higher prevalence of depression compared with men; however, this pattern appears to even out or even invert from ≥65 years of age, with women at lower risk compared with men.^23^ This has been primarily attributed to menopause, suggesting a hormonal influence.^24^ Psychosocial factors (e.g. personality characteristics, coping styles and perceived interpersonal problems) have also been postulated.^25^ Although higher age seemed to be protective against lockdown-related depression in women, this needs to be considered in the context of the wider study findings: significant increases in depression compared with pre-pandemic levels were observed across the cohort as a whole.

### Urban versus rural living

Living in urban (versus rural) areas was associated with a greater risk of depression during lockdown, with significance at trend for the whole sample, and significant (for women only) in the gender-stratified models. Pre-pandemic studies suggest that people living in urban areas are at higher risk of psychological distress, with increased risk likely attributable to both physical and social aspects of the urban environment. Physical aspects include less access to green areas,^26^ higher pollution and population density, and more physical threats (e.g. violence and crime).^27–29^ In the context of COVID-19, lockdown would likely have had more pronounced effects on those residing in urban areas, meaning extended periods of time spent indoors, with no access to a garden or green space, also limiting opportunities for physical exercise. Furthermore, those residing in urban areas report a weaker sense of belonging to their community and lower social support compared with those residing in rural areas;^30^ in older adults, a sense of community and social networks with neighbours are particularly important for well-being^31^ and reducing loneliness.^32^ Thus, lockdown would have further challenged the already weak sense of belonging and perceived social support for people living in urban areas, contributing to the effects seen here. Social participation is also an important contributor to healthy ageing,^33^ and is associated with decreased mortality and depression.^34^ Studies have found that older women tend to be more socially active compared with men,^35^ and are more likely to engage in organised community activities.^36^ During lockdown, community activities were prohibited, meaning that this opportunity for social participation was removed. Where people living in rural areas could potentially rely more on the local community and social engagement with neighbours to maintain some level of social participation, those living in urban areas might not have been able to. This could explain why urban living significantly affected women in particular: being more reliant on community activities for social participation, but having such opportunities removed from them, might have led to a greater impact on the mental health of women living in urban areas.

### Pre-existing depression

Baseline depression scores were entered into the models to control for pre-existing depression, and this was a significant predictor, regardless of gender. Pre-existing mental health issues have been associated with an increased risk of depression during lockdown.^11^ This trend likely derives from lockdown exacerbating pre-existing symptoms. Contributing factors include a pullback in community support, difficulty accessing treatment (many mental health services suspended their services, or switched to online/telephone appointments only) and reduced social contact with friends and family, as highlighted by a survey study of groups with lived experience of mental health issues.^6^

### Employment status

Likewise, employment status was a significant predictor; the effect was driven by those on long-term sick leave. This group showed a significantly greater increase in depression scores compared with those who were employed, unemployed or retired. Being in paid employment is associated with a lower risk of depression and better self-reported health,^37^ compared with being on long-term sick leave.^38^ As with pre-existing depression, lockdown conditions have exacerbated these effects. Having a long-standing illness also increased the risk of higher depression during lockdown, consistent with Robb et al^4^ who also found that subjectively reported poor health was a risk factor for depression and anxiety during lockdown. Generally, poor subjective health increases the risk of depression among older adults.^39^ In the context of the COVID-19 pandemic, because of the danger COVID-19 poses to those with pre-existing health conditions in particular, this likely served as a trigger for higher levels of worry and depression in these individuals.

### Living alone

Living alone was a significant risk factor for increased depression during lockdown in both genders; similar results were again found by Robb et al^4^ in their cross-sectional study in adults aged ≥50 years. Robb et al^4^ attributed this effect to loneliness: although loneliness was not assessed in this study, it seems likely that those living alone were differentially affected by the social isolation measures, removing opportunities for social contact and placing them at higher risk of loneliness and depression; associations between loneliness and depression have been reported across the lifespan.^6^

### Ethnicity

Interestingly, ethnicity effects were found such that non-BAME individuals reported greater change in depression compared with BAME individuals; gender-stratified analyses showed this to only be significant in women. This should be considered cautiously as BAME groups were underrepresented, comprising only 4% of the total sample. Other COVID-19-related studies found no influence of ethnicity on depression in adults across the lifespan.^13^ However, BAME groups also comprised relatively small proportions of those samples. This possibly suggests that either studies are underestimating COVID-19's effect on BAME groups because of lack of statistical power, or that BAME groups are at least equally resilient to mental health complaints during lockdown, compared with non-BAME groups. This is surprising, given that BAME groups have been disproportionately affected by COVID-19 in terms of mortality rates;^40^ the current finding that non-BAME individuals reported greater change in depression compared with BAME individuals goes against what one would predict, and merits further investigation regarding its validity and underlying causes.

### Smoking and alcohol use

Analyses found that smoking was not a significant predictor of change in depression for either gender. Alcohol consumption acted as a protective factor against the increased risk of depression in men only. In contrast, Robb et al^4^ reported that increased alcohol consumption in women contributed to depression during lockdown, with no effect in men. The current finding that alcohol consumption was protective in men might reflect greater social integration in men who drink. Studies have reported that in older adults, alcohol consumption is mostly socially motivated,^41^ particularly for men.^42^

### Socioeconomic factors

Finally, analyses revealed neither household income nor level of education significantly predicted change in depression. In contrast, other studies have identified education and income effects on risk of depression during lockdown, but in samples across the adult age range.^12–14^ However, these socioeconomic factors are perhaps less pertinent to adults ≥aged 50 years who (compared with younger age groups) have higher levels of accrued wealth and financial stability: 69% of our sample were retirees. Thus, socioeconomic status does not seem to have influenced depression risk in this age range; instead, negative effects seem to have been widespread regardless of education and income class.

### Strengths, limitations and future directions

The longitudinal nature of this study allowed temporal changes in depression to be considered, contrasting pre-lockdown with lockdown data. The overwhelming majority of comparable work has been cross-sectional in design; thus, the current study represents a valuable contribution to the literature. The study also utilised a robust outcome measure, the CES-D-SF, a seven-item screening tool for clinical depression, derived from the 20-item CES-D.^43^

Study limitations include a reliance on self-report data. In addition, the lack of full health data on participants meant additional health-related factors could not be incorporated in the models; further, measures of isolation, social connectedness and loneliness would have been beneficial. These would provide further insights into the social factors that influenced risk for depression during lockdown, but were not available. Likewise, information on the quantity (rather than just frequency) of alcohol use was not collected, which limits the interpretation of the alcohol-related effects observed in men. Also, although the longitudinal design is a key strength of this study, we only included data from two time points; future work assessing longitudinal change over a longer timeframe would be beneficial.

To summarise, this study identified risk factors that predicted change in depression in older adults as a result of COVID-19-related lockdown. Analyses suggested that depression in older adults significantly increased during lockdown compared with pre-pandemic levels in the UK. Risk factors included lower age, female gender, living alone and having a long-standing health condition. Gender-stratified analyses revealed effects unique to each gender: in women only, being younger and living in an urban area was associated with higher risk. More frequent alcohol usage acted as a protective factor, but only in men. These findings advance the literature by providing vital information regarding risk factors for poorer mental health during lockdown conditions, in an understudied age range; inferences are more robust compared with other studies because the current study utilised longitudinal data. Findings have important implications for risk stratification and intervention strategies, pointing to the demographic groups that should be prioritised for support, and those that are most at risk of poor mental health if a return to lockdown conditions is required in future. Specifically, the study findings suggest that women, particularly those that are younger and live in an urban area, are at highest risk. Thus, resources to combat COVID-19-related mental health issues might be best directed toward these individuals. Given the intense demands that the COVID-19 pandemic has placed on mental health services worldwide, this information is particularly important for allowing limited resources to be allocated more effectively.

## Data Availability

The data that support the findings of this study are publicly available at https://beta.ukdataservice.ac.uk/datacatalogue/series/series?id=200011. The data was accessed under project number 206540.
